# Immuno-inflammatory mechanisms in cardio-oncology: new hopes for immunotargeted therapies

**DOI:** 10.3389/fonc.2025.1516977

**Published:** 2025-03-13

**Authors:** Meiqi Miao, Xinxin Liu, Han Zhang, Hailong Dai

**Affiliations:** ^1^ Department of Cardiology, Kunshan Hospital of Chinese Medicine, Kunshan, China; ^2^ Postdoctoral Mobile Station, Heilongjiang University of Traditional Chinese Medicine, Harbin, China; ^3^ The Innovation Base, Mudanjiang Collaborative Innovation Center for the Development and Application of Northern Medicinal Resources, Mudanjiang, China; ^4^ Department of Cardiology, Yan’an Affiliated Hospital of Kunming Medical University, Kunming, China

**Keywords:** inflammatory response, cardio-oncology, cardiotoxicity, cardiovascular disease (CVD), biomarkers, immunotherapy

## Abstract

Cardio-oncology is an emerging interdisciplinary field concerned with cancer treatment-related cardiovascular toxicities (CTR-CVT) and concomitant cardiovascular diseases (CVD) in patients with cancer. Inflammation and immune system dysregulation are common features of tumors and cardiovascular disease (CVD). In addition to the mutual exacerbating effect through inflammation, tumor treatments, including immunotherapy, chemotherapy, radiation therapy, and targeted therapy, may induce immune inflammatory reactions leading to cardiovascular damage. Cancer immunotherapy is currently a new method of cancer treatment. Immunotherapeutic agents, such as immune checkpoint inhibitors (ICIs), chimeric antigen receptor T cell immunotherapy (CAR-T), mRNA vaccines, etc., can induce anti-tumor effects by enhancing the host immune response to eliminate tumor cells. They have achieved remarkable therapeutic efficacy in clinical settings but lead to many immune-related adverse events (irAEs), especially CTR-CVT. Establishing specific evaluation, diagnostic, and monitoring criteria (e.g., inflammatory biomarkers) for both immunotherapy and anti-inflammatory therapy-related cardiovascular toxicity is vital to guide clinical practice. This article explores the role of immune response and inflammation in tumor cardiology, unravels the underlying mechanisms, and provides improved methods for monitoring and treating in CTR-CVT in the field of cardio-oncology.

## Highlights

- CVDs are common complications of antineoplastic therapy, and they have shared risk factors with tumors.
- Increased inflammatory risk has a significant adverse effect on oncological cardiology-related markers, such as CTR-CVT.
- The “immune-inflammatory” mechanisms in CTR-CVT and tumor-associated cardiovascular complications are critical for patients’ safety, outcome, and prognosis.
- Inflammatory biomarkers can help assess the severity of CTR-CVT and reduce CVD incidence in cancer patients.
- Targeted therapy based on immune-inflammatory mechanisms holds promise for future treatment approaches in the field of cardio-oncology.

## 1 Introduction

Malignant tumors and cardiovascular diseases are two diseases with the highest morbidity and mortality rates, and both share common risk factors, such as smoking, obesity, diabetes mellitus, and hyperlipidemia. As healthcare improves and cancer treatment advances, the survival rate and lifespan of cancer patients increase. However, cardiovascular toxicities caused by cancer therapies have gradually emerged in recent years. Among cancer survivors, cardiovascular diseases are the second leading cause of morbidity and mortality after recurrent malignancies (1). Cancer therapy-related cardiovascular toxicities (CTR-CVT) has gained more and more awareness and attention, and has become the second leading cause of death among patients with cancer in addition to recurrence and metastasis. Therefore, an emerging cross-discipline has emerged, namely cardio-oncology (2). This field encompasses various research areas, including CTR-CVT, cardiovascular diseases (CVD) associated with tumors, shared risk factors between cardiovascular diseases and tumors, intervention strategies, and benign and malignant cardiac neoplastic lesions (3).

Tumors and CVD often coexist, with immune-inflammatory mechanisms acting as a link. The current view is that, on the one hand, activated immune cells secrete large amounts of inflammatory and other mediators in cancer (e.g., NF-κB, TNF-α, IL-1β, IL-6, etc.), affecting the function of distant organs, such as the heart. CVD have become widely recognized as complications of antitumor treatments, and cardiac cachexia is one of the most common cardiovascular complications of tumors. For instance, epidemiological evidence indicates that patients with breast cancer have a significantly increased risk of mortality from CVD following chemotherapy, surpassing the risk posed by the primary disease or recurrence (4). In addition, CVD stand as the primary cause of death among elderly cancer survivors. On the other hand, children with cancer and hypertension have a 12-fold higher risk of developing heart failure (HF) compared to survivors with normal blood pressure (5). A failing heart releases various factors into the bloodstream, including serotonin A1/A3 and other yet unidentified mediators, which can induce carcinogenesis (6). Additionally, an increasing number of studies have shown that increased risk of inflammation has a significant adverse effect on oncological cardiology-related markers such as CTR-CVT (7). Pan et al. (8) showed that atherosclerosis (AS), an underlying cause of CVD, is a tumor-like disease driven by smooth muscle cells, and ASCVD itself can be a carcinogenic condition.

**Figure 1:**
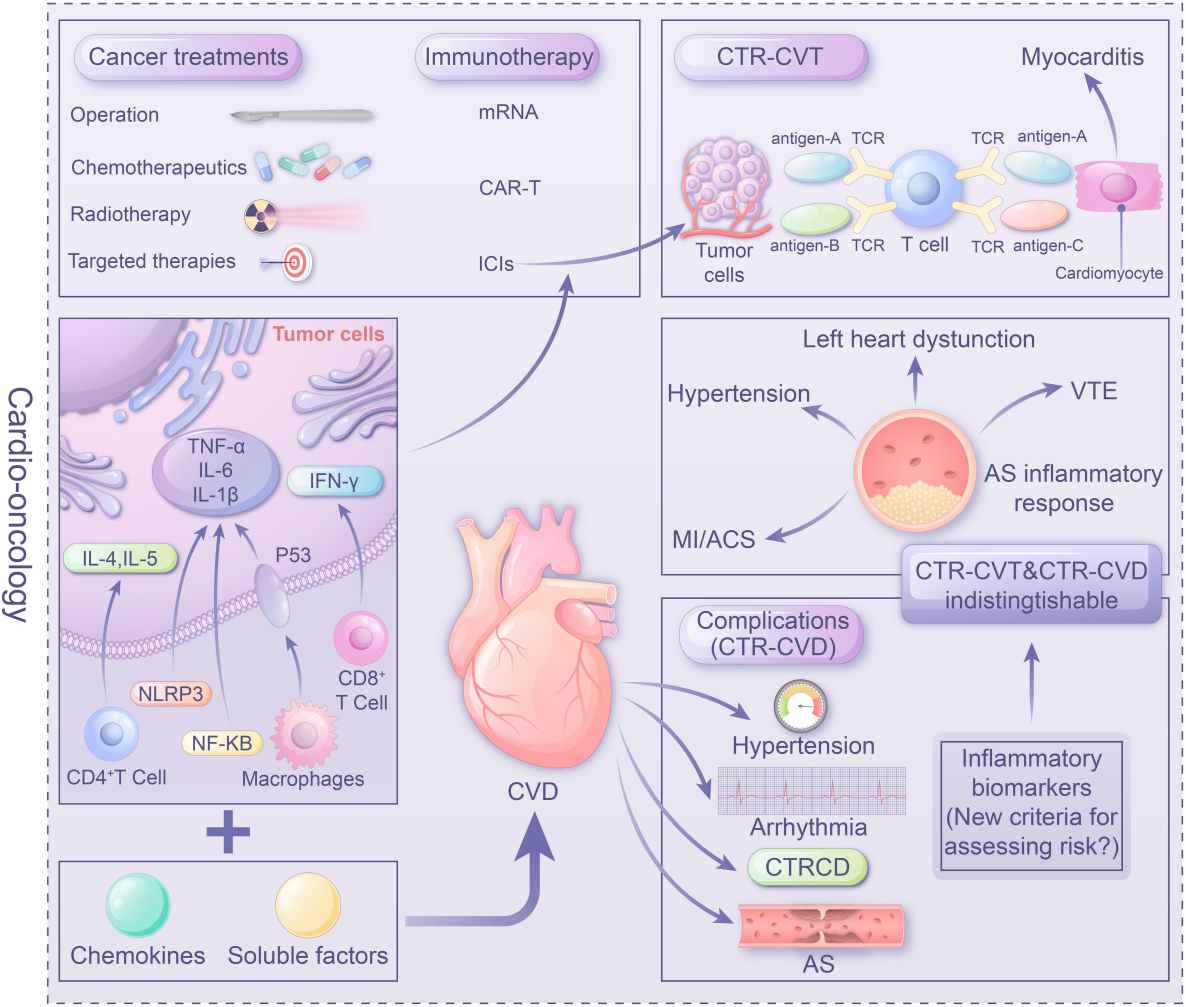
Center illustration: The role of the immune-inflammatory mechanisms in cardio-oncology as a link between tumors and CVD is crucial. Tumor-induced secretion of inflammatory mediators by immune cells (e.g. NF-κB, TNF-α) impacting heart function. Conversely, a failing heart releases factors promoting carcinogenesis. Understanding the role of inflammatory factors and mechanisms in CTR-CVT and tumor-related CVD is vital for managing interference between tumors and CVD, ensuring patient safety and treatment efficacy. Immune targeted therapies like ICIs, mRNA vaccines, and CAR-T provide a promising avenue in Cardio-oncology. However, unresolved issues, particularly establishing evaluation, diagnosis, and monitoring standards (e.g. inflammatory biomarkers) for immune therapy and anti-inflammatory therapy, warrant urgent attention in clinical practice.

In addition to the mutual exacerbating effect through immune-inflammatory mechanisms, tumor treatments, including immunotherapy, chemotherapy, radiation therapy, and targeted therapy, may induce immune inflammatory reactions leading to cardiovascular damage. Therefore, immune-inflammatory mechanisms serve as a shared mechanism between CVD and tumors. Understanding the relevant inflammatory factors and pathological mechanisms of immune inflammation in CTR-CVT and tumor-associated CVD is crucial for protecting patients against secondary complications and improving patient safety and treatment efficacy. This review focuses on the impact of ‘immune-inflammatory’ mechanisms in the development and progression of cardio-oncology, with the aim of providing new insights to guide future research into the pathogenesis of cardio-oncology and the quest for innovative therapeutic interventions (**Figure 1**).

## 2 Cancer treatment-related cardiovascular toxicities

In the 2022 ESC guidelines for cardio-oncology, CTR-CVT is classified as follows: chemotherapy-associated cardiovascular toxicity, targeted therapy-associated cardiovascular toxicity, immunotherapy-associated cardiovascular toxicity, endocrine therapy-associated cardiovascular toxicity, and radiotherapy-associated cardiovascular toxicity (2) (**Table 1**). Inflammatory response triggered by cancer treatments, including chemotherapy, radiotherapy, targeted therapy, and immunotherapeutic agents, can contribute to cardiovascular toxicity. These reactions include the release of inflammatory mediators, the production of autoantibodies, and the activation of immune cells. Additionally, individual factors, such as age, pre-existing cardiovascular diseases, and immune status, may affect the inflammatory response and cardiovascular toxicity.

**Table 1:** Cardiotoxicity caused by the tumor itself and by anti-cancer treatment.

Cardiotoxicity		CTRCD			Arrhythmia	Myocarditis	Coronary artery disease	Hypertension	Thrombosis and vascular embolism	Pericardial disease
		Heart damage	Cardiomyopathy	HF						
Tumor itself		✓	✓	✓	✓	✓				
chemotherapeutic drugs	Anthracyclines (Adriamyci)	✓	✓	✓	✓	✓				
	Cyclophosphamide (CTX)			✓		✓				✓
	Platinum metal complexes	✓			✓			✓		
	Drugs that act on cellular microtubules: TAX	✓	✓	✓	✓		✓	✓		✓
Targeted drugs	Antimetabolites: 5-fu				✓		✓		✓	
	HER2 or ErbB2 inhibitors (T-DXd)			✓	✓					
	VEGFI	✓		✓	✓			✓		
	EGFR-TKI (Osimertinib)			✓	✓					
	PIs (Kafizome)			✓			✓	✓	✓	
	BTK inhibitors			✓	✓					
	BCR-ABL kinase inhibitors			✓	✓		✓	✓	✓	
ICIs		✓		✓			✓			✓
CAR-T		✓		✓	✓					
Anti-CD20 therapy				✓	✓		✓			
mRNA vaccine					✓					✓
anti-inflammatory therapy				✓	✓		✓	✓	✓	
Endocrine therapy		✓			✓				✓	
Radiotherapy		✓		✓						

The symbol “✓” indicates the cardiac manifestations caused by different types of cardiotoxicity, respectively.

However, in addition to the cardiovascular toxicity caused by anti-cancer treatments, the tumor itself can also lead to cardiovascular toxicity. The study found that Hodgkin’s lymphoma survivors with an age of more than 50 had two times and five times more severe cardiovascular diseases of CTCAE 4.03 grade 3-5 than individuals without cancer and of the same age (9). Approximately 30% of Hodgkin’s lymphoma survivors over the age of 60 die from cardiovascular diseases, with cardiovascular mortality rates 3.8 times higher among the survivors of Hodgkin’s lymphoma, 2.7 times higher among the survivors of acute myeloid leukemia, and 1.7 times higher among the survivors of lung cancer (10). Therefore, understanding the mechanisms and management strategies for immune-inflammatory reactions and CTR-CVT is crucial for patient safety and treatment effectiveness.

### 2.1 Cardiotoxicity due to chemotherapeutic agents

**Anthracyclines (Adriamycin, etc.)**: As the drugs of choice for breast cancer chemotherapy, adriamycin has a strong antitumor effect. However, anthracyclines can cause myocardial damage through cumulative and dose-dependent effects (11). Anthracycline-induced cardiotoxicity (AIC) can manifest as a range of cardiac symptoms, including congestive heart failure, arrhythmias, myocardial infarction (MI), and other forms of cardiovascular diseases (12). Several clinical studies have found elevated levels of immune and inflammatory markers in patients with breast cancer who were treated with adriamycin (DOX). Another study showed that after 2 cycles of treatment with DOX, the plasma levels of macrophage migration inhibitory factor (MIF), which maintains cardiac homeostasis, were elevated in patients with breast cancer (13). Another clinical study on the use of DOX for treating breast cancer reported that compared to baseline, patients suffering from cardiac toxicity had significantly higher levels of IL-10, IL-1β, and NT-proBNP 7 days after completing treatment (14).

However, there are few studies on the role of the innate immune system in DOX-induced cardiotoxicity (DIC), such as the cytokine IFN-γ. It can inhibit DIC by targeting the AMPK/ACC axis and does not affect the anticancer effects of DOX. The activation of immune cells and neutrophil infiltration increases the risk of cardiotoxicity. High levels of plasma neutrophil extracellular traps (NETs) have also been linked to DIC (15). Bhagat et al. (16)found that DIC is associated with neutrophil infiltration and secretion of elastase (NE) in the heart, and inhibition of NE can significantly ameliorate DIC and attenuate cardiac damage. In addition, the crosstalk between neutrophils and M1 macrophages enhances Dox-induced inflammatory response and plays a key role in cell death (16). PI3K phosphatidylinositol 3-kinase γ (PI3K-γ) can prevent DOX-mediated cardiac insufficiency in the treatment of breast cancer via the autophagy pathway and simultaneously enhance the antitumor effects (17).

**Alkylating agents (cyclophosphamide, CTX, etc.)**: This alkylating agent is commonly used in combination chemotherapy for various solid tumors. CTX is well tolerated at low doses, and previous use of anthracyclines and mediastinal radiotherapy are considered to be contributing factors. Unlike anthracyclines, alkylating agents are more toxic at cumulative doses shortly after chemotherapy and can lead to heart failure, myocarditis, and pericarditis. High doses of CTX can affect myocardial capillaries (18). CTX at a therapeutic dose of 170-180 mg/kg induced a cardiotoxicity incidence of 7%- 28% and a mortality rate of 11% -43% (19). A multicenter open study showed that patients with HER2-positive limited breast cancer (BERENICE) treated with CTX experienced at least one decrease in left ventricular ejection fraction (20). It has been suggested that the possible cause of alkylating agent-based cardiotoxicity may be the alteration of membrane permeability and disruption of endothelial barrier function, leading to vascular injury, which in turn enhances the synthesis of inflammatory cytokines, such as TNF-α, IL-1β, and IL-6, similar to the inflammatory pathways associated with atherosclerosis (21, 22).

**Platinum metal complexes, such as cisplatin,** are widely used antitumor drugs worldwide, with precise efficacy, broad-spectrum anticancer effects, and relatively low prices. Platinum metal complexes may cause hypertension and increase cardiac load during hydration. Acute clinical syndromes associated with cisplatin include chest pain, palpitations, and occasional elevation of cardiac enzymes. Platinum drugs induce oxidative stress *in vivo*, thereby upregulating reactive oxygen species (ROS) levels and triggering an inflammatory response. Oxidative stress can damage cardiomyocytes, leading to apoptosis and dysfunction. ROS activates inflammatory pathways and releases pro-inflammatory cytokines (e.g., TNF-α, IL-1β, and IL-6), which further exacerbates myocardial injury (23). In a mouse model of cisplatin-induced cardiotoxicity, the nuclear factor Nrf2/heme oxidase-1 (HO-1) pathway was found to play a major role in suppressing oxidative stress and inflammation (23).

**Drugs that act on cellular microtubules (Paclitaxel):** Paclitaxel (TAX) is considered to be the most influential anticancer drug discovered by mankind to date. It can be used in the treatment of many types of cancers by acting on cellular microtubules. TAX can cause a series of cardiac adverse reactions, such as asymptomatic reversible bradycardia, blood pressure changes, arrhythmias, myocarditis, pericarditis, pericardial tamponade, and acute myocardial infarction (AMI), with the most common symptom being bradycardia (18). The cause of arrhythmias may be autonomic dysfunction of the heart and impaired cardiac conduction (24). However, there are limited studies on the cardiotoxicity mechanism of TAX, and since the cardiotoxicity of most anticancer drugs is due to the activation of the inflammatory/apoptotic/ROS pathway (25), we hypothesized that TAX induces cardiotoxicity through this pathway.

The use of nanoparticle delivery systems to carry chemotherapeutic drugs can effectively enhance therapeutic efficacy and prevent cardiovascular toxicity, and polyphenols (e.g., curcumin and flavonoids) can also alleviate cardiovascular toxicity of chemotherapy (26). However, due to individual differences and drug interactions, these two therapeutic means have low absorption, variable bioavailability and safety, and their mechanisms need further studies.

### 2.2 Targeted therapy-induced cardiotoxicity

Targeted antitumor drugs can lead to myocardial injury, arrhythmia, heart failure, hypertension, and vascular events through ion channel inhibition, inflammatory response, and vascular endothelial dysfunction. Anti-metabolites, such as 5-FU can induce ischemic syndromes, arrhythmias, and thrombosis, and increase cardiovascular risk in long-term use (27). Human epidermal growth factor receptor 2 (HER2 or ErbB2) inhibitors, such as detrastuzumab and trastuzumab, mainly cause reversible left ventricular insufficiency and tachycardia and exacerbate cardiotoxicity in combination with anthracyclines (28). Vascular endothelial growth factor inhibitors (VEGFIs) can induce heart failure and hypertension (29), affect hERG potassium channels, and increase the risk of QT prolongation (30). The epidermal growth factor receptor tyrosine kinase inhibitors (EGFR-TKI) ositinib can significantly increase the risk of heart failure (31), with a QT prolongation risk 49 times higher than that of other EGFR-TKIs (32). In addition, proteasome inhibitors, such as carfilzomib, cause heart failure, hypertension, and ischemic heart diseases (33, 34), mainly through NF-κB, AMPK inactivation, and autophagy regulation (35, 36). Bruton’s tyrosine kinase (BTK) inhibitors, such as ibrutinib, mainly regulate atrial fibrillation (37) and arrhythmias (38) through the PI3K-Akt pathway, leading to serious cardiac events (39). In contrast, BCR-ABL inhibitors, such as dasatinib, ponatinib, and nilotinib, can induce heart failure (40), atrial fibrillation (41), hypertension (42), and adverse vascular events (43) by inhibiting the VEGF signaling and inducing atherosclerosis (44).

Targeted therapies, as a revolutionary cancer treatment strategy, improve efficacy and minimize side effects by acting precisely on specific targets. Although targeted therapies have demonstrated unique advantages in improving efficacy and minimizing side effects, they still face several key challenges. First, drug resistance reduces treatment efficacy and leads to disease recurrence (45). To address this issue, combination therapy, as an effective strategy, can decrease the risk of drug resistance and improve efficacy by combining targeted drugs with other medications, such as chemotherapy and immunotherapy. Second, combination therapy can control side effects; certain targeted agents may affect normal cells and trigger toxicity while optimizing drug selectivity (e.g., antibody-drug couplers, nano-delivery systems), and individualized treatment can prevent side effects (46, 47). Third, cancer heterogeneity increases the difficulty of target screening; therefore, multi-omics analysis and artificial intelligence technology can improve the efficiency and accuracy of target discovery (48). Fourth, the complexity of individualized treatment, individual differences in tumors make it difficult to generalize the use of a single targeted drug. Genomic analysis, microenvironmental studies, and liquid biopsy can help precise treatment and dynamic adjustment of regimen. Finally, drug efficacy may decrease over time or drug resistance may emerge. Optimizing drug delivery and kinetic properties, combined with comprehensive treatment strategies, is expected to prolong efficacy and improve tolerance. Therefore, by improving the precision of targeted drugs, individualized treatment and long-term management can promote the development of targeted therapies.

### 2.3 Cardiotoxicity caused by immune checkpoint inhibitors

Cancer immunotherapy has emerged as a crucial clinical strategy for treating various solid tumors and hematologic malignancies (49). The application of ICIs significantly prolongs the overall survival of patients, and is regarded as a major breakthrough in cancer treatment. Immune checkpoints are expressed by various immune and non-immune cells to activate or deactivate the immune system. However, cancer cells can express these molecules to evade detection by the immune system. ICIs and monoclonal antibodies can block these immune checkpoints to reduce negative regulatory signals, enhance positive co-stimulatory signals, and modulate tumor antigen recognition by cytotoxic T lymphocytes. Programmed cell death protein 1 (PD-1), programmed death-ligand 1 (PD-L1), and cytotoxic T-lymphocyte-associated protein 4 (CTLA-4) are well-known immune checkpoints. Due to the marked therapeutic effects of ICIs in cancer patients, their immune-related adverse events (irAEs) have increasingly garnered attention (50–52). Cardiotoxicity is a more serious adverse reaction, and myocarditis, pericardial disease, heart failure, dyslipidaemia, MI, and cerebral arterial ischemia are the six major manifestations, the incidence rate is 3.2% ~19.3% (53). Palaskas et al. (54) proposed that PD-1/PD-L1 immune checkpoint inhibitor-induced myocarditis occurs due to the presence of common antigens in tumor cells and cardiomyocytes. One of these antigens is differently targeted by the TCR but homologous to the muscle antigen that serves as the tumor antigen, and the other one is a specific TCR targeting a different antigen. Drugs can similarly target the common antigen, causing myocardial injury and myocarditis. Liu et al. (55) found that deletion of the PCSK9 (a key protein regulating cholesterol metabolism) gene in mouse cancer cells significantly attenuated or prevented tumor growth in a cytotoxic T-cell-dependent manner, while significantly increasing PD-1 anti-cancer efficacy. Thus, due to their well-known safety profile, anti-PCSK9 antibodies may enhance the therapeutic efficacy of ICIs while reducing cardiotoxicity.

### 2.4 Cardiotoxicity due to endocrine therapy for breast cancer

Endocrine therapy for breast cancer can cause cardiotoxic manifestations such as dyslipidemia, lipodystrophy, metabolic syndrome, hypertension, heart failure, MI, and other cardiotoxic manifestations (1). Aromatase inhibitors (AIs) are widely used in adjuvant endocrine therapy for postmenopausal breast cancer, but may decrease estrogen levels, which in turn increase vascular endothelial dysfunction and increase the risk of AS (56, 57). In contrast, patients treated with the selective ER modulator tamoxifen suffer from a higher incidence of deep vein thrombosis compared to AIs (58). The emergence of cyclin-dependent kinase 4 and 6 (CDK4/6) inhibitors in recent years has brought new therapeutic options for HR-positive metastatic breast cancer. In clinical studies of CDK4/6 inhibitors, prolonged QT intervals and increased risk of sudden death were observed in patients treated with ribociclib (59). Besides, endocrine therapy may affect cardiovascular health by modulating the immune response. Studies have shown that low estrogen levels may aggravate chronic inflammation and activate T-cells and macrophages, thereby accelerating the development of atherosclerosis (60). Endocrine therapy not only affects the immune escape of cancer cells but also alters the immune microenvironment of the cardiovascular system. Studies have shown that tamoxifen may affect macrophage polarization and alter the inflammatory state (61), whereas aromatase inhibitors may affect cardiac immune homeostasis by modulating B and T cell function (62). Thus, endocrine therapy not only inhibits tumor growth by blocking hormonal signals but also modulates the immune response. However, the immune system also plays an important role in resistance to endocrine therapy through CD8+ T cells and the STING pathway (63). Researchers began the use of endocrine therapy in combination with ICIs to enhance anti-tumor effects to overcome drug resistance (64). Future studies will further reveal the immunomodulatory mechanisms and optimize the combination therapy strategy to improve efficacy and overcome drug resistance.

### 2.5 Radiotherapy-induced cardiotoxicity

Radiation therapy can activate several pathways, inducing oxidative stress, inflammation, microvascular dysfunction, myocardial injury, and fibrosis, collectively known as radiation-induced heart disease (RIHD) (65). Acute RIHD is mainly caused by radiation-induced acute inflammatory response in the heart (mainly myocardium) during radiotherapy (66). During acute injury, immune cells, such as neutrophils and macrophages, accumulate and secrete different cytokines that enhance the acute inflammatory response (67).

Chronic RIHD is mainly caused by chronic oxidative stress and free radical production, in which the cardiac fibroblast-induced cGAS-STING innate immune response pathway plays a key role in the inflammatory damage observed in chronic RIHD (68). Myocardial tissues and cells receive impacts on their function and blood supply with the advancement of the radiation process, which leads to the formation of myocardial fibrosis and cardiac hypertrophy, and ultimately exacerbating myocardial fibrosis and chronic cardiac injury (69). Studies have shown that breast cancer patients are at risk for serious ischemic events during radiotherapy, but lower mean heart dose (MHD) reduce the risk of major ischemic events (70).

In conclusion, the toxicities caused by cancer drugs are one of the primary obstacles to improving cancer treatment. A thorough understanding of anti-cancer treatment is crucial to detect, prevent, and provide targeted treatments.

## 3 Immuno-inflammatory mechanisms in CVD associated with antitumor therapy

In the 2022 ESC guidelines, cardiovascular complications associated with cancer treatment are categorized into 9 types: myocardial injury, heart failure, coronary artery disease, valvular disease, arrhythmias (particularly long QT syndrome), hypertension, thromboembolic disorders, peripheral vascular diseases, and stroke (2). Among them, myocardial injury and HF are the most severe.

### 3.1 CTRCD

CTRCD may occur due to direct cardiomyocyte damage, leading to primary cardiomyopathy. The most recent definition of CTRCD encompasses a wide range of cardiac manifestations, such as cardiac injury, cardiomyopathy, and heart failure, caused by cancer therapies, including chemotherapy, targeted therapy, immunotherapy, and radiation therapy (2). So far, the most extensively studied CTRCD is immune checkpoint inhibitor-related myocarditis, which has the highest fatality rate among all types of immune toxicities and remains a significant challenge for researchers. Immune inflammation may play a crucial role in developing immune checkpoint inhibitor-related myocarditis.

Endomyocardial biopsy (EMB) and post-mortem examinations revealed extensive infiltration of CD3^+^ T lymphocytes, including abundant CD8^+^ and CD4^+^ T lymphocytes, in the myocardial tissue of patients with ICI-associated myocarditis. Some patients also exhibit tissue infiltration of CD68^+^ macrophages, eosinophils, and rare CD56^+^ cells without evidence of antibody deposition (50). Additionally, there may be a certain degree of fibrosis in the myocardium, and the conduction system may also be involved. Furthermore, studies identified a shared high-frequency T lymphocyte receptor sequence in the myocardial and tumor tissues of such patients, suggesting that activated T lymphocytes following ICI therapy not only target tumor cells but also recognize shared antigens in the skeletal muscle and myocardium, thereby triggering autoimmune lymphocytic myocarditis (51). ICI-associated myocarditis is more common in patients with melanoma, but the exact mechanism remains unclear. In two reported cases of melanoma, during combined nivolumab and ipilimumab therapy, both patients developed myositis and rhabdomyolysis, accompanied by extensive infiltration of macrophages and T cells. Lymphocytes in the myocardium and tumor displayed the clonality of T cell receptor (TCR), indicating that the heart and the tumor may share antigens recognized by the same T cell clones (51).

### 3.2 Coronary artery disease

Tumor patients have several risk factors for developing acute coronary syndrome (ACS), with immune inflammation being a major risk factor. It has been reported that patients receiving ICIs may experience stable angina and ACS (71). The underlying pathological mechanisms involved in ICI-related MI are still unclear. Three possible hypotheses have been proposed in this regard. The first hypothesis suggests that ICI-related inflammation may affect atherosclerotic coronary artery plaques and trigger fibrous cap rupture, leading to AMI (72). Recent single-cell sequencing and mass spectrometry analysis of human atherosclerotic plaques have shown that T cells are the main immune cells in human AS lesions (73). Both CD4^+^ and CD8^+^ T cells are activated in the atherosclerotic plaques, which not only promotes the formation of AS lesions but also prompts the progression of the plaque to a vulnerable state, thereby increasing the risk of MI or ischemic stroke (51). PD-1 and PD-L1 are upregulated in myocardial ischemia and MI, increasing the functional molecules of CD4^+^ and CD8^+^ T cells and exacerbating AS in hyperlipidemic mice (73). Hypothesis 2: Transient ST-segment elevation caused by coronary artery spasm can occur during treatment with PD-1 inhibitor (pembrolizumab) (72). Hypothesis 3: T cells can induce coronary vasculitis (2). The lymphocyte/macrophage ratio was significantly higher in plaques of patients treated with ICIs (CD3/CD68 ratio) than in plaques of patients not treated with ICIs. Elevated CD3/CD68 ratio may be related to ICI-induced intra-plaque T-cell infiltration, reactivation of plaque T-cells, or increased T-cell-induced macrophage apoptosis (74).

Tumors and AS may be completely different diseases, but they share several pathophysiological features. For instance, some proteoglycans that bind strongly to low-density lipoprotein (LDL) are abundantly present in both AS regions and metastatic tumors. Tsumita et al. (75) demonstrated for the first time the mechanistic similarity between CVD and tumor progression. They found that highly metastatic tumor tissues accumulate large amounts of LDL, oxidized LDL (ox-LDL), and lectin-like ox-LDL receptor 1 (LOX-1). LOX-1 is an ox-LDL receptor that is highly expressed in tumor endothelial cells (TECs). The LOX-1/ox-LDL axis in TECs may lead to the formation of a highly metastatic tumor microenvironment by attracting neutrophils.

### 3.3 Arrhythmias

Cancer treatment can induce various types of arrhythmias, including sinus bradycardia, atrioventricular block, atrial fibrillation, and ventricular tachycardia. Tumor-related arrhythmias can be divided into primary and secondary arrhythmias. The former is caused by the effect of chemotherapeutic drugs on certain ion channels, and the latter is mainly secondary to myocardial lesions, cardiac insufficiency, hypertension, and other factors caused by antitumor drugs. It is not easy to differentiate between primary and secondary arrhythmias. Secondary arrhythmias are more common, with QTc of concern.

AF is the most common type of cancer-related arrhythmia and increases the incidence of postoperative arrhythmias in cancer patients (74). The pro-inflammatory state caused by cancer can trigger AF through atrial reorganization. The levels of circulatory CRP, an inflammatory marker in cancer patients, not only correlate with the presence of AF but also predict the risk of future AF (76). Many studies also confirmed a temporal association between the development of AF after tumor resection and the activation of proinflammatory factors, suggesting that inflammation may be an important factor in the development of postoperative AF. Other studies have shown that infiltration of immune cells and proteins that mediate the immune response in cardiac tissues and the circulatory system is associated with the development of AF (77, 78). Similarly, elevated levels of several inflammatory cytokines, such as C-reactive protein, TNF -α, IL-2, IL-6, and macrophage migration inhibitory factor (MIF), can be found in tumors and AF (79). The cause of ICI-associated arrhythmias is thought to be related to T-cell-mediated cytotoxicity (74). In the histopathology of one patient who developed conduction abnormalities after treatment with ICIs, patchy lymphocytic infiltration was seen in the sinus node and AV node (72).

### 3.4 Hypertension

Several chemotherapeutic agents impair vascular endothelium, sympathetic nerve activity, and renin-angiotensin system activity, which makes hypertension the most common comorbidity associated with tumors (66), and some scholars have proposed the new concept of “onco-hypertension”. Inflammation also links tumors to hypertension (80). Levels of inflammatory factors such as TNF-α, IL-6 and hsCRP are closely associated with the development and progression of hypertension. Angiotensin II (AngII)-induced hypertension increases the number of aortic monocytes/macrophages. Markers of aortic inflammation, such as vascular cell adhesion molecule-1, cyclooxygenase 2, and inducible molecules, and chronic low-grade inflammation, are associated with activation of NF-κB signaling and elevated levels of aromatase (the rate-limiting enzyme of estrogen biosynthesis) (76, 81) in adipose stromal cells of the breast. Aromatase regulates inflammatory mediators through several signaling pathways, including LKB1/AMPK, p53, HIF-1α and PKM2 pathways (76). A recent cohort study proposed that hypertension in middle-aged and young adults is strongly associated with the risk of death from colorectal cancer. They suggested that the renin-angiotensin-aldosterone system, which is responsible for the regulation of blood pressure, may play a role in biological processes such as cell proliferation, inflammation, angiogenesis, and tissue remodeling (82).

### 3.5 Thrombosis & vascular embolism

Patients with tumors have a significantly increased risk of pulmonary embolism, venous thrombosis, intracranial thrombosis, and arterial thromboembolism. Systemic chemotherapy (e.g., alkylating agents), anti-angiogenic drugs (e.g., bevacizumab), immunomodulators (e.g., thalidomide), hormonal therapies, and other therapies, such as immune-targeted therapies, have been shown to increase the risk of thromboembolism (1).

The mechanism of ICI-associated venous thromboembolic disease (VTE) has not been clarified so far, but some possible mechanisms have been suggested, including the expression of tissue factor-positive microparticles (TF-MPs) by T cells, increased abundance of myeloid-derived suppressor cells (MDSCs), and overproduction of inflammatory cytokines, etc. (83). SATO et al. (83) first proposed that ICIs activate T cells by blocking the immune checkpoint pathway, which further induces the production of F-MPs in PD-L1-overexpressing human peripheral blood CD14^+^ monocytes and impairs the function of the coagulatory system. In addition, it was found that both IL-8 and type 1 soluble vascular cell adhesion molecule (sVCAM-1), as important inhibitory cytokines that recruit and activate MDSCs, are significantly upregulated in the plasma of VTE patients than in the plasma of patients without VTE (84). In addition, MDSCs can promote the release of neutrophil extracellular traps (NETs) through CXCL8, thereby increasing the risk of thrombosis (84). Therefore, immunotherapy-associated VTE may be the result of IL-8 overexpression, and increased abundance of MDSCs promotes the release of NETs, which in turn triggers immune-mediated thrombosis.

On September 16, 2022, Health Canada alerted that Janus kinase (JAK) inhibitors (tofacitib, baricitinib, and upatinib) used to treat various chronic inflammatory diseases can increase the risk of major CVD (e.g., heart attack or stroke), tumors, deep vein thrombosis, pulmonary thromboembolism, serious infections, and death (85). This news also reveals that inflammation plays a role in tumor, CVD, and thrombosis.

### 3.6 Pericardial disease

The incidence is relatively low. Various reasons, such as infection, tumor invasion, radiotherapy, and chemotherapy, predispose tumor patients to pericardial disease, with acute pericarditis and pericardial effusion being the most common pericardial diseases in tumor patients (86). Analysis of patients with ICI-related pericardial disease revealed that none of the leukocytes (predominantly lymphocytes) in the pericardial effusion had cytological signs of malignancy (87). CT scan showed new-onset pericardial effusions and pericardial thickening. Also, cardiac MRI showed active pericardial inflammation (87). This may be due to the exposure of antigens shared by normal cells and cancer cells in cancer patients who underwent thoracic radiotherapy. ICIs may promote cytotoxic T cells to recognize antigens, which may induce pericardial inflammatory response and lead to pericardial effusion. In addition, Altan et al. (88) found high expression of CD68+ in the pericardial effusion of patients treated with ICIs and inferred that macrophage dysfunction is involved in the development of pericardial effusion.

### 3.7 Other diseases

**Valve Heart Disease (VHD):** The incidence is relatively low. Chemotherapeutic agents do not directly affect the heart valves, and valvular disease occurs usually secondary to pre-existing valvular disease, infective endocarditis, and left ventricular dysfunction. It has been reported that ICIs can lead to valvular dysfunctions, such as moderate-to-severe aortic valve insufficiency and mitral and tricuspid regurgitation by activating cytotoxic T cells. In addition, most of these lesions are accompanied by myocardial pathologies such as myocarditis or dilated cardiomyopathy (70). Baratchi et al. (89) found that constant compression of the stenotic aortic valve activates a large number of leukocytes, exacerbating inflammation and accelerating the progression of aortic stenosis. Transcatheter aortic valve implantation (TAVI) not only improves blood flow but also suppresses inflammation.

**Pulmonary Arterial Hypertension (PAH):** Venous thrombosis is the main cause of pulmonary vasculopathy in patients with tumors (90). Vascular remodeling and perivascular inflammatory cell (macrophage/lymphocyte) infiltration have been observed in human lung cancer tissues. PAH has been shown to be common in mouse models of lung cancer, in which chemokine production by tumor cells, together with perivascular inflammatory cell infiltration, is the main pathological mechanism (90). Zhang et al. (91), on the other hand, found for the first time that chronic thromboembolic pulmonary hypertension (CTEPH)/PAH may share similar characteristics and disease mechanisms with tumors, i.e., the conversion to a more pro-inflammatory IgG N-glycosome phenotype and the consequent decrease in the ability of IgG to inhibit chronic inflammation may be an important molecular mechanism in CTEPH. Recent studies have shown that PD-1/PD-L1 can inhibit helper T-cell responses through the PI3K/AKT/mTOR pathway and improve endothelial dysfunction in mice with hypoxia-induced pulmonary arterial hypertension (92).

**Heart Tumor:** Cardiac mucinous tumor, the most common cardiac neoplastic disease, is a less common and sporadic tumor that can lead to heart failure and systemic inflammatory symptoms and increase the risk of embolism. Intratumor inflammation and senescence appear to be involved. One of the major cellular mechanisms associated with tumor progression is autophagy, which is largely unknown in mucinous tumors. It was shown that the majority of mucinous tumors highly express autophagy markers LC3B and p62/sequestosome 1. LC3B expression was positively correlated with PD-L1 and CD163 expression, while LC3B was negatively correlated with CD8, CD20, CD138, and CD117 expression (93).

## 4 Targeted therapy in cardio-oncology based on immuno-inflammatory mechanism

The coexistence of cancer and CVD makes treatment complex, as treating one condition may adversely affect the other. However, immune-targeted therapies have emerged as a promising approach in both cancer treatment and certain CVD conditions that are refractory to conventional therapies. This highlights the broad potential of immune-targeted treatments in the field of cardio-oncology, offering new avenues for treatment.

### 4.1 Anti-inflammatory therapy

Atherosclerosis (AS) underlies the development of most CVDs, and since the introduction of the “inflammation theory” in AS by Russel Ross in 1990, more and more studies have confirmed that inflammation is a key driver of AS and its complications. Various cells (monocytes, macrophages, vascular endothelial cells, vascular smooth muscle cells, and T lymphocytes, etc.) and related inflammatory cytokines (C-reactive protein (CRP), interleukin IL-6, and IL-1, etc.) are involved in inflammatory signaling pathways and development of AS (94). At the molecular level, the formation of NLRP3 inflammatory vesicles in macrophages is a key step in the spread of inflammation, and the NLRP3/IL-1/IL-6/hypersensitive CRP (hs-CRP) classical inflammatory pathway is closely associated with the increased risk of atherosclerosis (95). hs-CRP, an important biomarker of inflammation, can directly reflect the inflammation to a certain extent; therefore, several guidelines at home and abroad recommend hs-CRP ≥2mg/L as a risk factor for CVD (96, 97). Many scholars conducted large-scale clinical trials to verify the role of inflammation in CVD [**Table 2** (98–107)].

**Table 2:** Large clinical trials on inflammation and CVD.

Time (year)	Trial name	Characteristics	Study Objects	Inflammatory factors	Reference
2010	Justification for the Use of Statins in Prevention: an Intervention Trial Evaluating Rosuvastatin (JUPITER)	Double-blind RCT	Rosuvastatin	hs-CRP	(98)
2017	Canakinumab Anti-inflammatory Thrombosis Outcome Study (CANTOS)	Double-blind RCT	Canacizumab	IL-1β	(29)
2018				IL-6	(30)
2019	Colchicine Cardiovascular Outcomes Trial (COLCOT)	Double-blind RCT	Colchicine	NLRP3 inflammasome	(31)
2020- 2021	Low-dose colchicine(LoDoCo, LoDoCo2)	Double-blind RCT	Colchicine	NLRP3 inflammasome	(32–34)
2020	CIRT	Double-blind RCT	Methotrexate(MTX)	IL-6, hs-CRP	(35)
2023.3	CLEAR Outcomes Trail	Double-blind RCT	Statin drugs	hs-CRP	(36)
2023.11			Beperidinic acid		(37)

For the first time, the landmark study of anti-inflammatory therapy for AS, the Canakinumab Anti-Inflammatory Thrombosis Outcome Study (CANTOS), demonstrated that anti-inflammatory drugs targeting IL-1β (such as canakinumab) can reduce the incidence of adverse cardiovascular events in patients with MI by lowering lipid levels (108). Similarly, Colchicine Cardiovascular Outcomes Trial (COLCOT) and LoDoCo2 trial demonstrated that colchicine can reduce the risk of cardiovascular events in patients with chronic coronary artery disease and recent MI who are already receiving standard therapy (94, 109–111). They also indicated that specific inflammatory pathways are involved in human ASCVD, and highlighted the role of NOD, LRR, and nod-like receptor protein 3 (NLRP3) inflammasome-associated pathways as effective therapeutic targets for ASCVD.

Despite advances in IL-1β-neutralizing therapies, reliance on a single cytokine inhibition may not be sufficient to fully address inflammation in cardiovascular diseases. Recently, several cytokine receptor blockers have been progressively studied in the cardiovascular field, such as anti-TNF-α monoclonal antibodies (e.g., infliximab and adalimumab), which have reduced the incidence of cardiovascular events and improved the symptoms of heart failure in several clinical studies (112). However, the widespread use of TNF-α inhibitors needs more clinical data, especially when the risk of infection is high (113). In several clinical trials, anti-IL-6 receptor monoclonal antibodies (e.g. tocilizumab) have been shown to attenuate the inflammatory response by inhibiting IL-6 activity, which subsequently mitigates cardiovascular diseases (114). However, the multiple biological roles of IL-6 also suggest the possible risk of immunosuppression with this treatment, and further studies are needed to focus on its long-term efficacy and safety. Neutralizing antibodies against IL-18 (e.g., toleragen) have shown the potential to reduce cardiovascular inflammation and improve cardiac function in preclinical studies; however, relevant clinical data are limited and research in this direction is promising (115). Vascular endothelial growth factor (VEGF) plays an important role in neovascularization and inflammatory responses, especially in AS plaque formation and the rupture of unstable plaques (116). The progression of AS can be reduced by inhibiting VEGF activity. Although this strategy is mainly used in the treatment of cancer, it is also a research hotspot in the cardiovascular field, especially in patients with a high risk of AS.

However, anti-inflammatory therapy reduces the “residual inflammation risk” and also reduces the effect of anti-cancer therapy, and can even lead to some cardiovascular toxic effects. Anti-infectives, non-steroidal anti-inflammatory drugs (NSAIDs) and other commonly used drugs that reduce inflammation, such as statins and metformin, have been shown to reduce cancer risk. However, NSAIDs can simultaneously affect the cardiovascular system through several mechanisms, increasing the risk of MI, heart failure, hypertension, and other cardiovascular diseases (117). Therefore, they can be combined with nanoparticle-carrying drugs to enhance the therapeutic effect and avoid cardiotoxicity at the same time. They have not yet been widely used in the clinic due to the high cost or adverse effects. There are still unmet clinical needs for anti-inflammatory therapy, which necessitates new anti-inflammatory targets and drugs to protect patients against cardiotoxicity while reducing the effectiveness of anti-cancer treatment.

### 4.2 Inflammatory biomarkers

Inflammatory responses are involved in the pathogenesis of oncological heart disease; therefore, inflammatory biomarkers can serve as cardiovascular toxicity markers after cancer treatment. They can be observed before and after oncological heart disease (mainly for cardiovascular toxicity risk assessment, treatment regimen assessment, cardiotoxicity monitoring protocols, and post-hospital follow-up management) to determine the severity of oncological heart disease. Therefore, it is urgently needed to establish a unified cardiovascular assessment, diagnosis, or monitoring standard for anti-inflammatory therapy to guide clinical practice. Currently known markers of immune inflammation are summarized below:

**Inflammatory cytokines** such as NF-κB, IL-6, IL-1β, and TNF-α are indicators of inflammation and can be used to assess the severity of cancer-associated myocardial damage (5).

**Inflammatory markers, such as** VCAM1, sST2, and adiponectin, can be used as early indicators of decreased cardiac function in patients with breast cancer following antitumor treatments (can be used for determining tumor type and staging) (118).

**Neutrophil-to-lymphocyte ratio (NLR), Platelet-to-lymphocyte ratio (PLR), Monocyte-to-lymphocyte ratio (MLR), Neutrophil-to-eosinophil ratio (NER):** Many studies have demonstrated a close association between the combination of NLR and PLR, and the development and prognosis of cardiovascular diseases. The combination of NLR and PLR showed a better predictive efficacy for cardiovascular adverse events than individual markers (119). MLR can be used to assess the risk of CTRCD in patients with breast cancer. An increased NER during iRC indicates a more severe disease and has a prognostic value for overall mortality. Higher NER and NLR values suggest a more severe iRC (120, 121).

**Inflammatory response(SII) Index:** SII is a novel biomarker of inflammation that integrates platelet, neutrophil, and lymphocyte counts, offering a higher predictive value than NLR and PLR (122). It provides a more accurate and comprehensive assessment of immune and inflammatory responses. Initially proposed by HU et al. (123), SII reflects the severity of systemic inflammation in patients with cancer. However, recent findings have linked SII to the severity and prognosis of certain CVD (124). A recent cross-sectional study on the SII index in the general population of the United States demonstrated a U-shape correlation between the SII index and all-cause mortality, cardiovascular disease-related mortality, and tumor-related mortality in patients with CVD. Therefore, the SII index can serve as a predictor of all-cause mortality, CVD mortality, and tumor-related mortality in patients with CVD (125). Thus, maintaining the SII index within an optimal range may effectively reduce the incidence of cardiovascular events in tumor patients.

**Myeloperoxidase(MPO):** Changes in MPO can respond to changes in cardiotoxicity after antitumor therapy; the combination of MPO and troponin I (TnI) improves risk prediction for cardiotoxicity (126–129).

**hs-CRP:** CRP is a potential biomarker for assessing the risk of overall tumor and 12 site-specific tumors (130). hs-CRP levels can independently predict all-cause cardiovascular mortality risk in the general population.

The use of inflammatory biomarkers can help the early diagnosis of MI and myocarditis, which may contribute to timely treatment and improve the prognosis of patients. Researchers have found the results of the test to inhibit the inflammatory pathway to cut off the nutrient supply and growth signals of the tumor, thereby effectively inhibiting tumorigenesis and targeting the tumor heart disease after treatment (131). The emergence of multi-omics technology, artificial intelligence, and machine learning technology in recent years has brought new opportunity for precision medicine, exploring new biomarkers for precision treatment.

### 4.3 Immune checkpoint inhibitors

Recently, ICIs have emerged as a significant advancement in cancer treatment. These antibodies primarily inhibit immune checkpoints, thus enhancing the ability of effector T lymphocytes to recognize and eliminate tumor cells. ICIs stimulate an augmented systemic antitumor immune response (132). Cardiac adverse effects of ICIs include fulminant myocarditis, myocardial-pericardial inflammation, heart failure, arrhythmias, and MI. Accumulating evidence suggests that immune checkpoint molecules PD-1 and PD-L1 play a crucial role in maintaining cardiac homeostasis. Moreover, histopathological exploration is challenging, making it difficult to explore the pathophysiological mechanisms85and establish diagnostic approaches (133). Preclinical evidence indicates that PD-L1/2 and CTLA-4 blockade or knockout on endothelial cells and cardiomyocytes, can enhance cardiac infiltration of immune cells (134, 135), leading to fatal myocarditis. In cases of suspected ICI-related myocarditis, high-dose steroids are the preferred first-line treatment, and commonly used immunosuppressants serve as the second-line medications. Therefore, protecting the cardiovascular system during ICI-based antitumor therapy is a research hotspot needing further investigation. Currently, the treatment of myocarditis caused by ICIs is mainly stopping ICIs and injecting high doses of corticosteroids. When the above two treatments cannot be implemented or are ineffective, a combination of mycophenolate mofetil and high-dose methylprednisolone or emerging drugs, such as tofacitinib (a JAK-STAT inhibitor), tolizumab (an IL-6 receptor inhibitor), abasicap, intravenous immunoglobulin, and anti-thymocyte globulin, etc., can be helpful. However, there are individual differences in treatment effects, which needs further studies (26).

### 4.4 CAR-T cell therapy

Chimeric antigen receptor T cell immunotherapy (CAR-T cell therapy) combines the specificity of chimeric antigen receptors with the T cell immune response to selectively kill malignant tumor cells (136). After the recognition of tumor antigens, CAR-T cells release pro-inflammatory cytokines IL-1, IL-6, IFN- γ, and TNF-α to induce a cytotoxic response (137). Tissue damage is usually caused by the activation of cytokine release syndrome (CRS) and may be directly associated with fatal adverse events. Clinical studies have shown that cardiovascular toxicity associated with CAR-T is characterized by hypotension (5-30%), left ventricular dysfunction (5-10%), pulmonary edema (4-5%), arrhythmias (4-8%), and heart failure (1-6%) (138–141). Currently, cell-based therapies, especially CAR-T cell therapy, are widely utilized for treating tumors, exhibiting promising results, which have received widespread attention. In addition, it also has potential therapeutic effects on cardiac injury, and mouse studies have shown that this method can effectively reverse myocardial fibrosis and improve function after injury (142). Rurik et al. (143) encapsulated mRNA in bubble-like micro-LNPs and injected them into mice, similar to mRNA vaccines. The encapsulated mRNA molecules were captured by T cells, converting them into CAR-T cells targeting myocardial fibroblasts, thereby improving cardiac function in mice with heart failure. CAR-T cells treat myocardial fibrosis after myocardial injury. Even by hindering only a small percentage of disease-causing fibroblasts, CAR-T cells can effectively restore myocardial function. Thus, in the future, CAR-T cell therapy can be combined with other modalities of immunotherapy to take advantage of its highly precise and personalized potential in the field of cardio-oncology.

### 4.5 mRNA vaccines

Recently, the preventive use of tumor vaccines has evolved into therapeutic applications. Tumor mRNA vaccines induce or enhance the body’s anti-tumor immune effects by activating the body to generate an immune response, which in turn induces or enhances the body’s anti-tumor immune response (144). Therapeutic vaccines for CVD are bioformulations composed of specific antigenic epitopes of targeted molecules and carriers. They induce the production of specific antibodies against these self-epitopes and exert therapeutic effects on the targeted molecules (145). Currently, mRNA vaccine development primarily focuses on the treatment of tumors and infectious diseases, while research on cardiovascular diseases has been confined to hypertension, diabetes, and atherosclerosis. There are only a few case reports of mRNA vaccine-induced cardiac injury, including myocarditis and pericarditis (146), which suggests the potentially unfavorable T-cell response of mRNA vaccines. The balance of the immune effect and safety aspects still needs further studies. However, it is undeniable that mRNA vaccines will play a prominent role in cardio-oncology in the future.

### 4.6 Anti-CD20 therapy

CD20 plays a key role in the development, differentiation, and activation of B cells. It is an important target for the treatment of B cell malignancies. Anti-CD20 immunotherapy can specifically target B cells and has minor effects on other types of cells. B cells can recover after stopping treatment and do not affect the patient’s humoral immunity (147). Anti-CD20 immunotherapy is rapidly developing. In addition to traditional monoclonal antibodies (mAbs), there are antibody-drug couplings (ADCs), bispecific antibodies (BsAbs), and CAR-T. Studies have shown that B cells can either play a direct role through differentiation into plasma cells and secretion of antibodies, or an indirect role through antigen presentation and release of cytokines or chemokines to promote an antitumor response. They possess regulatory effects, but more studies are needed to unravel the specific antitumor mechanisms. In a real-world study, two CD20 inhibitors, ocrelizumab and ofatumumab, were significantly associated with various adverse cardiovascular events, particularly coronary artery disease, heart failure, and atrial fibrillation (148).

In addition to their role in cancer, accumulating evidence indicates that B cells play an equally important role in AS and HF progression. Clinical studies have shown that treatment with rituximab (RTX), a human-mouse chimeric monoclonal antibody targeting CD20, can improve endothelial function and reduce arterial wall thickness and arterial stiffness (149–151). Anti-heart autoantibodies can cause cardiac injury directly through functional or cytotoxic effects or indirectly through the formation of antigen-antibody complexes and complement activation, leading to inflammatory cardiac injury (152). Experimental studies have shown that mature B lymphocytes mobilize inflammatory monocytes into the heart after AMI in mice, leading to increased infarct size and deterioration of cardiac function. These findings suggest that RTX possesses a protective effect (153). A recent trial provided preliminary evidence of the safety and feasibility of acute single RTX infusion in patients with ST-segment elevation MI (STEMI) (154). In addition, in patients with dilated cardiomyopathy, an elevated abundance of TNF-α-secreting B cells positively correlated with the cardiac fibrosis marker type III procollagen (155). RTX has been successfully used in selected patients with chronic inflammatory cardiomyopathy and improved survival in patients undergoing transplantation and suffering from antibody-mediated rejection (156). However, it has also been shown that RTX accelerates the development of allograft vasculopathy after cardiac transplantation (157). A phase II clinical trial is currently evaluating the safety and efficacy of RTX in patients with stable grade III/IV HFrEF (158). Thus, the role of CD20+ B cells in cancer and CVD reveals their complexity in the pathogenesis of both diseases. Their pathological effects are time-dependent and context-dependent, depending on the microenvironment and immune response, and may even be associated with the NLRP3/IL-1β pathway.

## 5 Conclusion and outlook

Immuno-inflammatory mechanisms play a pivotal role in the development of CTR-CVT and CTR-CVD. In this paper, we summarized the latest research progress in the prediction of tumor-associated CVD and inflammatory markers in CTR-CVT, but there is still a lot of work to be done:

1. Although some clinical trials have demonstrated that inflammation and immune system dysregulation are common mechanism between tumors and CVD, there is a lack of additional clinical data to support this.
2. The mechanisms linking these novel inflammatory markers, such as MPO, high-sensitivity troponin, and hs-CRP, and anti-tumor therapy-associated cardiovascular toxicity has not been fully investigated.
3. It should be investigated whether these inflammatory markers can be combined and developed into a systematic scoring system for risk stratification of patients with tumor-associated CVD and CTR-CVT for prevention and early diagnosis and treatment.
4. Although studies on anti-inflammatory therapy for AS have made some progresses in reducing adverse cardiovascular events and mortality in patients with ASCVD, there is still a lack of appropriate clinical trials to clarify their indications; thus, anti-inflammatory therapy still has a long way to go in the prevention and treatment of tumor-associated CVD and CTR-CVT.
5. The discovery of various predictors of CTR-CVT has played a key role in achieving therapeutic efficacy in anti-tumor cardiology. Many of the emerging therapeutic strategies mentioned in the paper, such as nano-bearers, can be combined with several anti-cancer treatment modalities to reduce the incidence of CTR-CVT. They have not yet been widely used in the clinic due to the high cost or adverse effects. There are still unmet clinical needs for anti-inflammatory therapy, which necessitates new anti-inflammatory targets and drugs to protect patients against cardiotoxicity while reducing the effectiveness of anti-cancer treatment.

Therefore, targeted therapies based on immune-inflammatory mechanisms may become an extremely promising new avenue for treating CTR-CVT and cardiovascular complications associated with oncologic therapy, and research in this area has the potential to improve treatment outcomes and prognosis of patients with tumor cardiology in the future.

## Glossary

ACS: Acute Coronary Syndrome
AF: Atrial fibrillation
AS: atherosclerosis
CAR-T: chimeric antigen receptor T cell
CHF: congestive heart failure
CTLA-4: Blocking cytotoxic T-lymphocyte-associated protein 4
CTLs: cytotoxic T lymphocytes
CTRCD: cancer therapy-related cardiac dysfunction
CTR-CVT: cancer therapy-related cardiovascular toxicity
CVD: cardiovascular disease
ESC: European Society of Cardiology
ICIs: immune checkpoint inhibitors
irAEs: immune-related adverse events: iRCs, ICI‐related cardiotoxicities
HF: heart failure
LNPs: lipid nanoparticles
MI: myocardial infarction
MLR: Monocyte-lymphocyte ratio
MPO: myeloperoxidase
NF-κB: nuclear factor-κB
NER: neutrophil-to-eosinophil ratio
NLR: neutrophil-to-lymphocyte ratio
NLRP3: nod-like receptor protein 3
PD-1: Programmed cell death protein 1
PD-L1: PD-1 and its ligand 1
PLR: platelet-to-lymphocyte ratio
QTc: corrected QT interval
ROS: reactive oxygen species
SII: systemic immune inflammation
sST2: soluble suppression of tumorigenicity 2
TAMs: tumor-associated macrophages
TILs: tumor-infiltrating lymphocytes
Th1: T helper cell 1
Th2: T helper cell 2
TnI: troponin I
VCAM-1: vascular cell adhesion molecule 1
